# Evaluation of Permeate Quality in Pilot Scale Membrane Distillation Systems

**DOI:** 10.3390/membranes9060069

**Published:** 2019-06-05

**Authors:** Alba Ruiz-Aguirre, Juan A. Andrés-Mañas, Guillermo Zaragoza

**Affiliations:** 1 Dipartimento di Ingegneria, Università degli Studi di Palermo (UNIPA), viale delle Scienze, Ed. 6, 90128 Palermo, Italy; 2 Department of Chemical Engineering, Universidad de Almeria—CIESOL, 04120 Almería, Spain; juanantonio.andres@psa.es; 3 CIEMAT—Plataforma Solar de Almeria, Ctra. de Senés s/n, 04200 Tabernas, Almería, Spain

**Keywords:** desalination, brine treatment, membrane distillation, pilot scale, permeate quality, membrane filtration, high salinity

## Abstract

In this work, the salinity of permeate obtained with membrane distillation (MD) in pilot scale systems was analyzed. Experiments were performed with three different spiral-wound commercial modules, one from Solar Spring with 10 m^2^ surface membrane area and two from Aquastill with 7.2 and 24 m^2^. Intermittent operation meant that high permeate conductivity was measured in the beginning of each experiment, which was gradually decreasing until reaching a constant value (3–143 µS·cm^−1^ for seawater feed). The final quality reached did not depend on operating conditions, only the time it took to reach it. This can be because the permeate flux dilutes the minimal feed leak taking place through pinholes in the membranes. Larger feed leak through the membrane was observed when operating in vacuum-enhanced air-gap MD configuration (V-AGMD), which is compatible with this explanation. However, for the increase of feed leak with salinity (up to 1.8 M), a conclusive explanation cannot be given. Pore wetting due to crystallization is discarded because the high permeate quality was recovered after washing with distilled water. More studies at higher salinities and also at membrane level are required to investigate this.

## 1. Introduction

Seawater desalination has become the main option used to alleviate the problem of water scarcity in coastal regions [1]. Out of all the different desalination processes, membrane technologies are the most widely used throughout the world. Among them, reverse osmosis (RO) is the most studied and industrially implemented method because of its economic competitiveness compared to other processes [2,3,4]. However, the problems related to the discharge of concentrated brines into the environment have not yet been resolved and are gaining increasing relevance [5]. In recent years, science and technology of membranes have improved in trying to find solutions to these environmental problems in order to achieve the goal of zero liquid discharge (ZLD) [6,7]. Additionally, this concept offers the opportunity of recovering valuable materials from desalination brines after a process of membrane crystallization [8,9,10,11,12]. Since pressure-driven membrane processes such as RO cannot treat concentrated brines because of their high osmotic pressures, thermal desalination technologies must be used [13,14]. A non-isothermal membrane process such as membrane distillation (MD) appears as a suitable solution. MD has been proposed to treat aqueous solutions of high salt concentration derived from other desalination processes, for producing more water improving the quality of the RO product water, and for reducing the volume of waste in order to achieve ZLD [8]. The main challenge of MD technology in this context is the possibility of irreversible pore wetting of the membrane resulting in a considerable decline of MD performance and ultimately the subsequent discard of the membrane. However, not many studies have been performed with MD for treating high salinity solutions, and the majority of them have been carried out at a laboratory scale. Experimental works with high concentration aqueous solutions of NaCl have been carried out using air-gap MD [15,16], direct contact MD [17,18] and vacuum MD [19], but they have all been done at a very small scale, typically for surface membrane areas of 0.014 m^2^ or lower. Also for the treatment of RO brines with MD, experiments have been mostly performed at lab scale, using DCMD [20,21] and VMD [22], with membrane areas of 0.014, 0.04 and 0.16 m^2^, respectively. Even when coupling membrane distillation with crystallization [11,22,23,24], the scale of the experiments is usually too small for extrapolating to commercial modules. As many authors state, experiments at pilot scale are needed [25]. Few studies are reported, Minier-Matar et al., [26] compared two commercial MD technologies at pilot scale (membrane areas of 6.4 and 4.6 m^2^, respectively) for treating brines from thermal desalination (70 g/L total dissolve solids), obtaining a high quality distillate (< 10 µS·cm^−1^) with the first one (vacuum-enhanced 4-effect MD configuration). Duong et al [27] studied the behaviour of a pilot MD system from Aquastill (membrane area 7.2 m^2^) for the treatment of RO brine from coal seam gas (CSG) produced water. The permeate conductivity was reported to be 500 µS·cm^−1^, which is higher than that obtained in lab-scale studies. Winter et al [28] characterized the performance of the Oryx 150 module from Solar Spring (membrane area 10 m^2^) for different feed salinities (0–1.8 M) without focusing on the quality of the permeate. However, they reported larger values than in lab-scale experiments, as also done by Ruiz-Aguirre et al., [29] using a similar module but with simulated seawater (0.6 M). Finally, Schwantes et al [30] studied an AGMD module adapted with an active draining of the air gap by low pressure air blowing using a range of concentration on the feed between 0 g·kg^−1^ and 240 g·kg^−1^. Although the study was focused on the characterization of flux, GOR and thermal efficiency, permeate conductivity values were shown. Comparing results without and with blower, the drainage of the air gap with pressurized air, improved the overall average of the quality of permeate and the increase of permeate conductivity with higher feed salinity was significantly mitigated. There are not many other studies of MD at pilot-scale where the permeate quality has been analyzed in depth. 

In this work, a Solar Spring module and two different Aquastill modules were used to study the quality of permeate considering different feed concentrations in a range including high salinity, to test whether these commercial modules are suitable for industrial implementation. These modules have been fully characterized in terms of permeate production and energy efficiency [30,31,32], but extensive information on permeate quality has not been provided yet.

## 2. Materials and Methods 

In this study, three different MD commercial spiral wound modules were evaluated: Oryx 150, AS7, and AS24. The Oryx 150 module was designed by Solar Spring (Freiburg, Germany) in collaboration with Fraunhofer Institute for Solar Energy Systems ISE (Fraunhofer ISE, Freiburg, Germany), while the AS7 and the AS24 modules were built by Aquastill company (Sittard, Netherlands). The former was operated in permeate-gap (PGMD) configuration, while the latter two were operated both in air-gap (AGMD) and vacuum-enhanced air-gap (V-AGMD) configurations. Table 1 summarizes the main characteristics of the three modules. 

To carry out the experiments, synthetic solutions were prepared as feed, using marine salts obtained from the natural saltworks of Cabo de Gata (Almería, Spain). Feed solution for the different concentration tested (in a range from 0.6 M to 2.4 M) was prepared dissolving these salts in demineralized water. Demineralized water was produced from brackish water using RO and electrodeionization. The chemical properties of demineralized water were: conductivity: 2 µS·cm^−1^, pH: 6.3 and turbidity 0.1 NTU. The dissolved total carbon (DTC) was 1.33 mg·L^−1^ and dissolved inorganic carbon (DIC) was 0.42 mg·L^−1^. 

**Figure 1:**
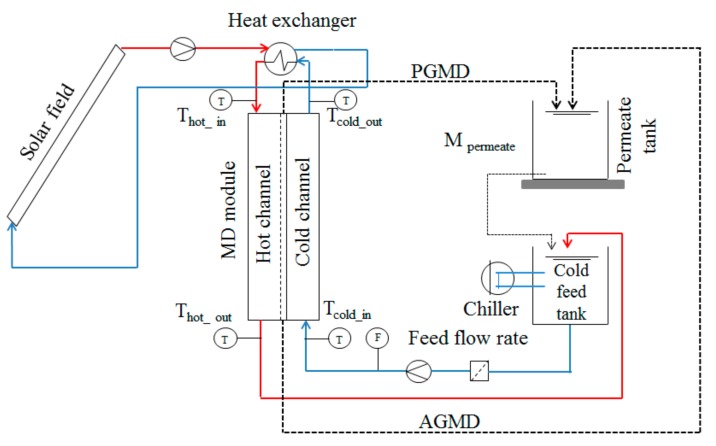
Process flow diagram of the membrane distillation (MD) operation.

The operation of the commercial MD modules consisted of pumping firstly the feed water from the feed tank into the cold channel (see Figure 1). The feed, pre-heated with the latent heat of condensation, was then additionally heated up in a heat exchanger connected to a solar thermal field, and then it flowed along the hot channel, ending up in the feed tank for recirculation. Permeate was also returned to the feed tank to keep a constant salinity, but three samples were collected every 15 min for measuring its conductivity. Since residual heat of both currents overheats the feed tank, the feed water was cooled with a compressor chiller before entering the module again. More details are given elsewhere for the specific operation of the SolarSpring [33] and the Aquastill [32] modules in this pilot plant.

In the Oryx 150 module, all the inlets and outlets were located on the top of the module, so the module was left filled with the feed between the tests. In the AS7 and the AS24 modules, the cold inlet channel and the hot outlet channel were placed at the bottom of the module. Therefore, to avoid the emptying of the module when tests were not performed, the circulation of the feed through the module was not interrupted, only reduced and continued without the application of heat.

In this study, a series of experiments with a different hot inlet temperature (T_hot_), cold inlet temperature (T_cold_), feed flow rate (F), and feed salinity (S) were considered. In total, a large number of experiments were performed along a large period of time (total operation time of the pilots exceeded 300 h). Table 2 summarizes the combination of the operating conditions in the experiments carried out with the different modules. The flow rate setpoints were achieved by using a variable frequency drive connected to the circulation pump. The changes were done gradually, and the hydraulic pressure inside the module was monitored continuously to prevent it from exceeding the maximum value given by the module manufacturers (700 mbar in the case of SolarSpring and 600 mbar in the case of Aquastill), with the control system acting accordingly. Thus, the hydraulic pressure on the surface membrane was kept more than five times below the nominal liquid entry pressure of the membranes (4 bar in the case of SolarSpring, 3.8 bar in the case of Aquastill). In the AS7 and the AS24 modules, experiments were carried out in standard AGMD and V-AGMD configuration. In the latter case, the suction of air from the gap resulted in absolute pressure in the gap channel varying between 150 mbar and 250 mbar. Experiments with each combination of operating conditions were carried out three times to guarantee the statistical validity of the results.

The permeate quality was analyzed in terms of the conductivity, measured with conductivity meter Portavo 902 (version COND, Knick), conveniently calibrated for the appropriate salinity ranges. In order to remove the influence of temperature in these measurements and facilitate their comparison, temperature reference was set to 20 °C. Samples of around 40 mL each 15 min were taken and measured twice. In the case of the Aquastill V-AGMD system, sampling time cannot be fixed, because permeate was discharged discontinuously by the system with a frequency that was very dependent on operational conditions. The conductivity meter provides directly the value of electrical conductivity of the solution referred to 20 °C. Finally, conversion from electrical conductivity (EC) in mS·cm^−1^ to concentration (c) in g·L^−1^ was made (see Appendix A). 

Salt Rejection Factor (SRF) was calculated with the following expression:

(1) $$\mathrm{SRF}\left(\%\right)=\frac{c_{f}-c_{p}}{c_{f}}\cdot 100,$$

where $c_{f}$ is the feed concentration in g·L^−1^ and $c_{p}$ is the permeate concentration also in g·L^−1^.

The membrane leak ratio, representing the ratio of the feed that passes through the membrane, was calculated following Equation (2). A normalized membrane leak ratio (to membrane surface area) was also calculated (see Equation (3)).

(2) $$\mathrm{Membrane}\;\mathrm{leak}\;\mathrm{ratio}\;\left(\%\right)=\frac{\mathrm{Permeate}\;\mathrm{flow}\;\mathrm{rate}\;\left(L\cdot h^{-1}\right)}{F\;\left(L\cdot h^{-1}\right)}\cdot \frac{C_{p}\;\left(g\cdot L^{-1}\right)}{C_{f}\;\left(g\cdot L^{-1}\right)}\cdot 100,$$

(3) $$\mathrm{Normalized}\;\mathrm{membrane}\;\mathrm{leak}\;\mathrm{ratio}\;\left(\%\cdot m^{-2}\right)=\frac{\mathrm{Membrane}\;\mathrm{leak}\;\mathrm{ratio}\;\left(\%\right)}{\mathrm{Surface}\;\mathrm{Membrane}\;\mathrm{Area}\;\left(m^{2}\right)},$$

## 3. Results and Discussion

### 3.1. Evolution of the Quality of Permeate along the Operation with the Oryx 150 Module

**Figure 2:**
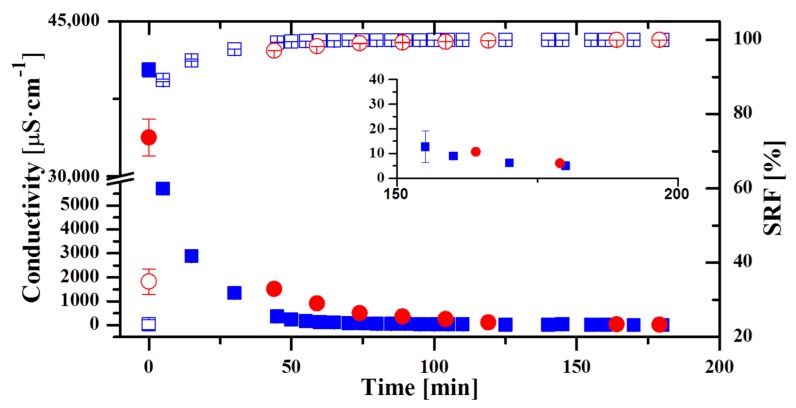
Permeate conductivity and Salt Rejection Factor (SRF) obtained in two typical days of operation with the module Oryx 150. Blue squares: conductivity of day 1; red circles: conductivity of day 2; blue empty squares: SRF of day 1; red empty squares: SRF of day 2.

Figure 2 shows the evolution of the conductivity (left) and SRF (right) of permeate obtained during two different days of operation with the Oryx 150 module for a feed salinity of 0.6 M. The curve represented with blue squares illustrates one day when it was operated with two different operating conditions. During the first 25 min, the variables were adjusted to the desired ones (F = 500 L·h^−1^, T_hot_ = 70 °C and T_cold_ = 30 °C). Later, it operated during 75 min in those conditions. After that, another experiment started increasing T_hot_ by 5 °C with respect to the previous experiment. These conditions were also kept during 75 min after a transition period of 25 min. The conductivity of the first permeate sample obtained was 40,300 µS·cm^−1^ (equivalent to a salt concentration of 27 g·kg^−1^), that would correspond to a SRF of 23.4%. However, after only 5 min, the conductivity decreased considerably (600%) to reach 6000 µS·cm^−1^ (3 g·kg^−1^). After 50 min from the first permeate, the conductivity was 239 µS·cm^−1^, already below the taste threshold established by the World Health Organization (WHO): for sodium chloride in the range 0.2–0.3 g·kg^−1^, which corresponds to a conductivity of 400–600 µS·cm^−1^ [34]. This WHO standard was taken only as a reference, not implying that permeate produced in MD can be drinkable (as a matter of fact, for that purpose the permeate would generally need remineralization). The corresponding SRF for this value was higher than 99%. The conductivity continued to decrease, reaching a final value of 5 µS·cm^−1^ (0.003 g·kg^−1^) corresponding to a SRF value close to 100% (99.99%). The curve represented with red circles corresponds to another day when the module was operated with two different conditions. As in the previous case, the first 25 min were used to adjust the operating variables. After that and during 75 min, the module was operated at F = 400 L·h^−1^, T_hot_ = 60 °C and T_cold_ = 20 °C. Later, the T_hot_ was changed to 65 °C keeping the rest of the variables during 75 min constant. The conductivity of the first sample was 33,750 µS·cm^−1^ (21.7 g·kg^−1^). As in the previous case, the quality of the first permeate was low (equivalent SRF would be 35%). The conductivity decreased to 1515 µS·cm^−1^ (1 g·kg^−1^) 45 min after obtaining the first permeate. After 45 min, the conductivity reached a value of 350 µS·cm^−1^ (0.18 g·kg^−1^). In this day, a very low value of conductivity was also reached finally (4 µS·cm^−1^ (0.002 g·kg^−1^)). The trend of the two curves was the same, namely, a high conductivity in the first permeate and then a decrease to lower values throughout the daily operation. The high conductivity when starting the operation is often explained by the formation of crystals inside the pores, when the operation is stopped [35]. These crystals would be washed as permeate is produced when restarting the operation, therefore the improvement of the permeate conductivity along the operation. However, crystallization inside the pores would cause permanent damage, as is the case for crystallization fouling [36,37]. The fact that excellent permeate quality is obtained consistently after several months of operation when this initial bad permeate quality takes place every day, makes this explanation unlikely. Another possibility is the leaking of feed solution through possible pinholes existing in the membrane. When the operation ends, the flux of vapor is interrupted and only the small passage of liquid feed through the defects of the membrane is collected in the permeate channel. When the operation restarts, permeate has a high salinity because it contains the feed liquid. Since the fraction of feed liquid that passes through the membrane is low (membrane leak ratio of 10^−5^%), as more permeate is produced this leak is diluted in the condensation channel and the permeate conductivity decreases.

Similar results were reported in other studies [28,35]. However, in the study carried out by Winter et al. [19] with a similar module, the conductivity level decreased faster at the beginning of the experiment than in this work. This could be due to different operating conditions. As illustrated in Figure 2, the final permeate quality did not depend on the operating conditions, as expected from the results obtained in other studies [38]. The influence of the operating conditions was on the time necessary to reach conductivity below the taste threshold. When operating conditions drove to higher permeate production, the time needed was lower. For example, in the first day, the necessary time was 50 min; in the second day, 89 min were needed. This was due to the fact that in the first day the permeate production was greater than in the second one (Table 3). The reduction of the permeate production (40%) from one day to another was almost proportional to the increase of the time (44%) necessary to achieve a good permeate quality (WHO taste threshold). The objective of this work was not to analyze the permeate flux in the modules, which was already studied in other publications [31,32]. However, it is worth stressing that the low values shown in Table 3 are a common feature in all commercial modules, and is explained because of the lower driving force as a consequence of the higher heat transfer through the membrane along the longer channels compared to lab-scale modules. Fluid velocity in the feed channel of the Oryx 150 module was between 0.08 m·s^−1^ and 0.11 m·s^−1^ (corresponding Reynolds numbers 155 and 230, respectively) [31].

**Figure 3:**
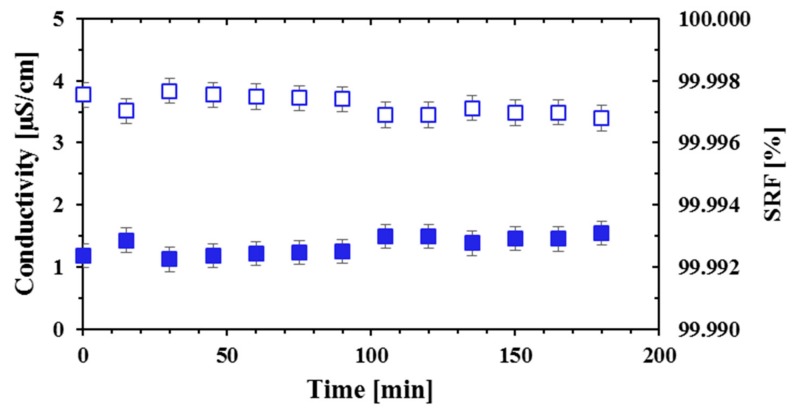
Permeate conductivity and SRF obtained with the module Oryx 150 after cleaning with demineralized water at 80 °C. Blue squares: conductivity, blue empty squares: SRF.

Figure 3 shows the permeate conductivity obtained in an experiment performed after the module had been left filled with demineralized water subsequently to a cleaning with this water at 80 °C. The experiment was carried out with F = 400 L·h^−1^, T_hot_ = 80 °C and T_cold_ = 20 °C. Permeate conductivity was lower than 2 µS·cm^−1^ (0.001 g·kg^−1^) since the beginning and throughout all the experiment, being SRF practically 100%. Therefore, cleaning and leaving the module filled with demineralized water was enough to avoid the high conductivity at the beginning of the experiment. The experiment started shortly after filling the module with the feed solution. Although leak of the feed through the pinholes may have occurred, this was not accumulated before permeate production took place from the vapor flux and so, the conductivity spike was not observed. This helps validate the hypothesis that the high salinity at the beginning of the experiment was due to the leak of liquid feed through membrane defects as pinholes. 

### 3.2. Evolution of the Permeate Quality along the Operation with Aquastill Modules

**Figure 4:**
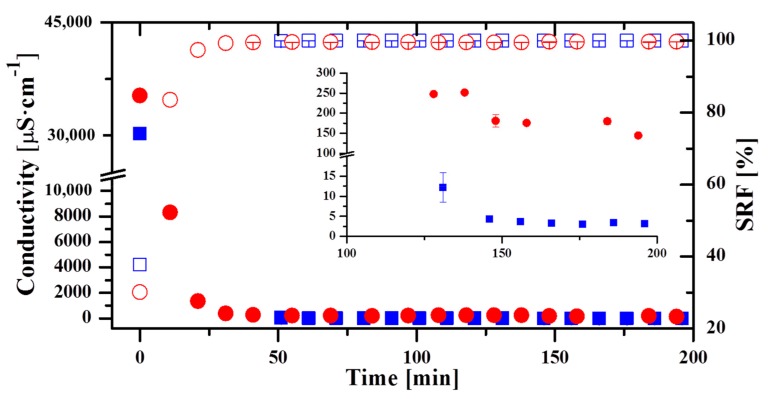
Permeate conductivity and SRF in an operation day with the AS7 module (blue squares and blue empty squares respectively) and the AS24 module (red circles and red empty circles respectively) both in AGMD configuration.

Figure 4 shows the evolution of the conductivity (left) and SRF (right) for a typical day of operation with the AS7 (squares) and the AS24 (circles) modules. These two days are representative of the permeate quality obtained throughout the operation with these modules for a salinity of 0.6 M. In both curves, the initial time corresponds to the moment when the first permeate was collected. In the AS7 module, the curve corresponded to one day in which two different operating conditions were tested with their corresponding stabilization time. Specifically, it was first operated with F = 400 L·h^−1^, T_hot_ = 70 °C and T_cold_ = 20 °C. After that, F was increased up to 500 L·h^−1^. Regarding the AS24 module, operating conditions tested the reference day were firstly F = 500 L·h^−1^, T_hot_ = 80 °C and T_cold_ = 20 °C, later F was increased to 600 L·h^−1^, and finally T_hot_ was varied to 70 °C. At the beginning, as in the Oryx 150 module, the conductivity was very high; though later the values were lower than the taste threshold established by WHO. In the AS7 module, after 50 min the permeate conductivity was 46 µS·cm^−1^ and in the AS24 the taste threshold was reached after 40 min. The conductivity of the samples continued to decrease, though the minimum value reached with the AS24 module was greater than that of the Oryx 150 and the AS7 modules. While in the AS7 module the conductivity dropped to a value of 3 µS·cm^−1^ (SRF practically 100%), the minimum permeate conductivity in the AS24 module was 140 µS·cm^−1^. The corresponding SRF was 99.7%. Although this value was lower than that obtained in the Oryx 150 and the AS7, it is still higher than that obtained with other desalination technologies (between 95% and 98% for SWRO [39]). As in the case of the Oryx 150 module, the final permeate quality did not depend on the operating conditions. The worse final permeate quality obtained in the AS24 could be due to the greater membrane surface and therefore higher probability of having defects on the membrane as pinholes. The membrane leak ratio was about 10^−4^% for the AS7 module and 10^−3^% for the AS24 module. Moreover, the AS7 module had larger permeate flux than the AS24 module (Table 4) [32]. The worse permeate quality in the AS24 module limits its use in certain applications. For example, the conductivity of permeate required in the refrigeration circuits of diesel engines or in the preparation of urea to reduce NO_x_ emissions of a thermal power plant has to be lower than 100 µS·cm^−1^ [40]. 

### 3.3. Effect of the Feed Salinity on the Permeate Quality

**Figure 5:**
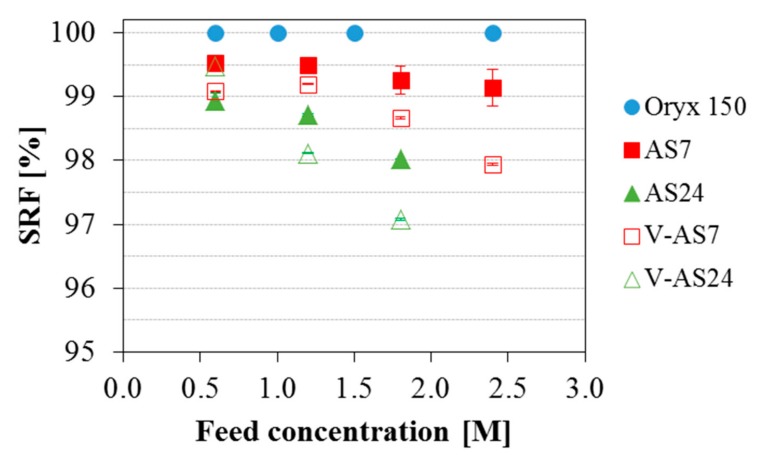
SRF obtained with the Oryx 150, the AS7 and the AS24 modules (in AGMD and V-AGMD configurations) for different initial feed concentrations. T_hot_ = 80 °C, T_cold_ = 25 °C and F = 1100 L·h^−1^ (AS modules) and 600 L·h^−1^ (Oryx 150).

**Figure 6:**
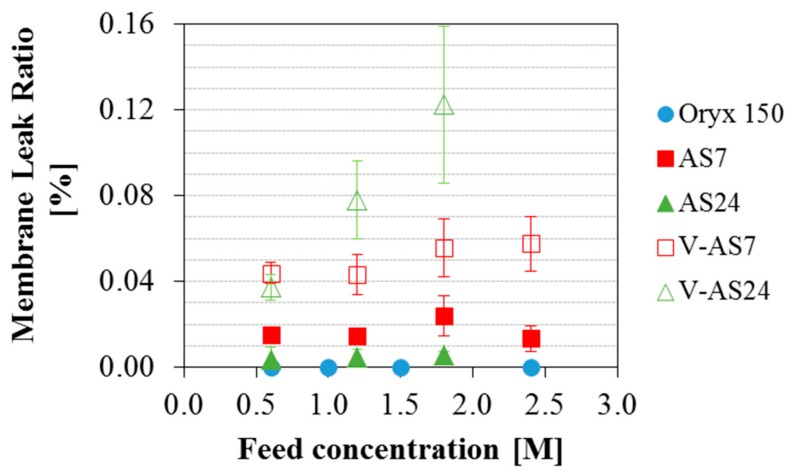
Membrane Leak Ratio obtained with the Oryx 150, the AS7 and the AS24 modules (in AGMD and V-AGMD configuration) for different initial feed concentrations. T_hot_ = 80 °C; T_cold_ = 25 °C; F = 1100 L·h^−1^ (AS modules) and 600 L·h^−1^ (Oryx 150).

**Figure 7:**
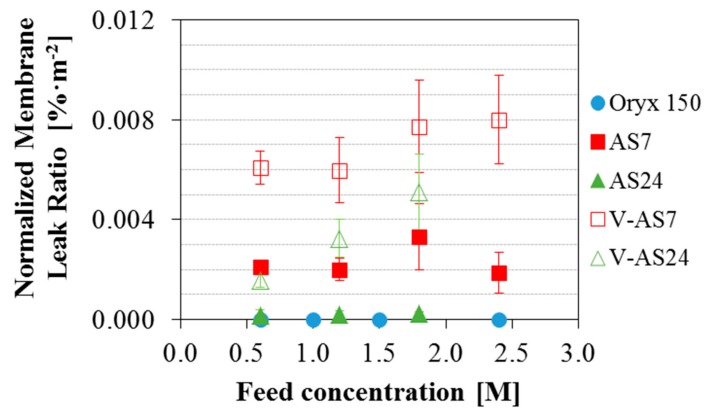
Normalized Membrane Leak Ratio obtained with the Oryx 150, the AS7 and the AS24 modules (in AGMD and V-AGMD configuration) for different initial feed concentrations. T_hot_ = 80 °C; T_cold_ = 25 °C; F = 1100 L·h^−1^ (AS modules) and 600 L·h^−1^ (Oryx 150).

The previous tests were done for feed salinity equivalent to seawater. Once established that the operating conditions (temperatures and flow rate) do not affect the final permeate quality obtained with each module, the focus of the work was put on the analysis of the influence of the feed salinity on the different modules. Figure 5 shows the SRF for the three MD commercial modules analyzed in this study considering both configurations (AGMD and V-AGMD) in the AS modules for different initial feed concentrations. Operating conditions were nominal (optimized for maximum permeate production) for all feed concentrations. For the AS7 and the AS24 modules T_hot_, T_cold_ and F were 80 °C, 25 °C and 1100 L·h^−1^ respectively, and for the Oryx 150 module, the only change was that F was 600 L·h^−1^. The Oryx 150 module showed excellent separation performance with a SRF always above 99.99% for the different feed concentrations tested. The two Aquastill modules tested produced a good quality of permeate for a feed concentration of 0.6 M in both AGMD and V-AGMD configurations. For feed concentration of 1.2 M, only the AS7 module in AGMD configuration showed a SRF according to the WHO standard value. For the rest of feed concentrations, AS modules showed a SRF lower than that, and worsening as the feed concentration increased. When both configurations (AGMD and V-AGMD) were compared, the application of vacuum in the gap affected negatively the permeate quality. Moreover, the effect of the vacuum was slightly more pronounced in the AS24 module than in the AS7. For example, for feed concentration 1.8 M, while in the AS7 module the SRF reduction reached 0.6%, in the AS24 module it amounted to 1%. The application of vacuum reduces the mass transfer resistance in the gap, favouring the passage of liquid feed through the pinholes. This effect was slightly stronger in the AS24 module, possibly due to its larger surface membrane area. Also, the permeate quality decreased as the salinity of the feed (and therefore that of the leak) increased. This effect was also shown by Schwantes et al. [30]. To better assess this matter, the membrane leak ratio was calculated and compared for the different modules (Figure 6). The maximum value was around 0.12% in the AS24 module when operating in V-AGMD configuration for the highest salinity. For this module, reliable tests at feed salinity larger than 1.8 M were not available. In standard AGMD configuration, the values of the membrane leak ratio were always less than 0.024%. The fact that larger leak appeared for the high salinity feeds could indicate the presence of higher crystallization fouling, but as discussed before this would have a non-reversible effect which is not observed. For elucidating this matter, more detailed studies are needed analyzing the membranes behaviour with high salinity, something that can only be done to the full extent at a laboratory scale where membranes can be isolated and analyzed completely. Another interesting issue shown in Figure 6 that requires further investigation is that the membrane leak ratio was larger for the AS24 module than for the AS7 in V-AGMD (as expected by the larger membrane surface, as discussed in Section 3.2) but not in AGMD. When normalizing by the membrane surface area (Figure 7), however, it was confirmed that the AS7 module is actually the one with the higher leak. This suggests a difference in the membrane quality between the two modules used in these experiments (perhaps a larger ratio of pinholes per area in the AS7), or some other unaccounted effect influencing both modules differently. To assess the significance of this difference, an investigation comparing more modules of the same kind (AS7 and AS24) would be needed.

## 4. Conclusions

Based on the results obtained from this work, it can be stated that MD demonstrates technological robustness for desalinating feed solutions with seawater concentration, because commercial modules analyzed in this study produced permeate of very good quality (SRF greater than 99%) during the entire period of testing (a large number of experiments performed along more than 300 h of operation), under very different operating conditions of T_hot_, T_cold_ and F. However, intermittent operation generated a poor permeate quality when the process was restarted. This can be because liquid feed passes through possible membrane pinholes while no production of permeate occurs. When the operation is initialized, the permeate production dilutes the initial conductivity because the vapor flow is several orders of magnitude greater than the feed leak through the membrane. When the module was kept with demineralized water after cleaning, permeate had a very high quality from the beginning. The recovery of the permeate quality discards crystallization fouling, which would cause an irreversible deterioration of the membrane hydrophobicity. Permeate quality decreased with the increase of feed salinity, especially in the case of the Aquastill modules. The PTFE membrane used by the SolarSpring module showed much better retention than the LDPE membrane used by Aquastill, although in the worst case, the maximum leak through the membrane in the latter was never more than 0.12% of the feed. A conclusive reason cannot be given for this worsening of permeate quality with salinity. The fact that the membrane leak for the AS7 module was slightly larger than for the AS24 module, when their membrane is supposedly the same kind, suggests that other effects are present or the membranes properties can suffer some variation in the performance. More studies of these modules and the membranes behavior with high salinity feeds are needed to investigate this. 

## Figures and Tables

**Table 1:** Characteristics of the membrane distillation (MD) commercial modules used in this study.

Characteristics	Oryx 150	AS7/AS24
Configuration:	PGMD	AGMD and V-AGMD
Channels length:	7 m	1.5 m/5 m
Channels thickness:	3.2 mm	2 mm.
Membrane surface area:	10 m^2^	7.2 m^2^/24 m^2^
Membrane material:	active layer: PTFE; support: PP	active layer: LDPE ^1^
Active layer thickness:	70 µm	76 µm
Active layer pore size:	0.2 µm	0.3 µm
Active layer porosity:	80%	85%
Support layer thickness:	280 µm	-
Support layer porosity:	50%	-
Gap thickness:	1 mm	0.88 mm
Vacuum pressure:	-	150–250 mbar abs

^1^ no support.

**Table 2:** Operating conditions tested on the three commercial MD modules.

Variable (Unit)	Module	Range of Operation
T_evap_ (°C)	All modules	60–80
T_cond_ (°C)	All modules	20–30
F (L·h^−1^) *	Oryx 150	400–600
	AS7, AS24	400–1100
S (M) **	Oryx 150	0.6–1; 1–1.5; 1.5–2.4
	AS7	0.6–1.2; 1.2–1.8; 1.8–2.4
	AS24	0.6–1.2; 1.2–1.8

* The highest limit of F was established by the maximum hydraulic pressure allowed. ** S range was chosen considering recommendations of the module manufacturers.

**Table 3:** Permeate production with the operating conditions of the cases shown in Figure 2.

Day	F (L·h^−1^)	T_hot_ (°C)	T_cold_ (°C)	P_flux_ (L·h^−1^·m^−2^)
1	500	70	30	1.5
2	400	60	20	0.9

**Table 4:** Permeate production with the operating conditions of the cases shown in Figure 4.

Module	F (L·h^−1^)	T_hot_ (°C)	T_cold_ (°C)	P_flux_ (L·h^−1^·m^−2^)
AS7	500	70	20	2.7
AS24	600	80	20	1.1
AS24	600	80	20	1.3
AS24	600	70	20	1.1

**Figure A1:**
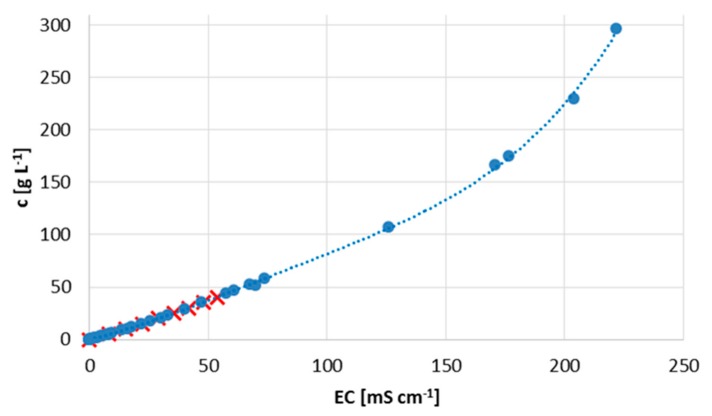
Salt concentration as a function of electrical conductivity. Blue dots: NaCl aqueous solution. Red crosses: seawater.

## Acronyms

AGMD	Air gap membrane distillation
CSG	Coal seam gas
DIC	Dissolved inorganic carbon
DTC	Dissolved total carbon
GOR	Gained output ratio
LDPE	Low density polyethylene
MD	Membrane distillation
MVC	Mechanical vapour compression
PP	Polypropylene
PTFE	Polytetrafluoroethylene
RO	Reverse osmosis
SRF	Salt rejection factor
V-AGMD	Vacuum-enhanced air gap membrane distillation
WHO	VWorld health organization

## Nomenclature

C EC	Concentration (g·L^−1^) Electrical conductivity (mS·cm^−1^)
F	Feed flow rate (L·h^−1^)
T	Temperature (°C)
S	Salinity (M)

## Subscripts

cold	Cold channel
f	Feed
hot	Hot channel
p	Permeate

## Appendix A

The exact composition of the marine salts used in this work is not available. Typically, marine salts have a proportion of NaCl close to 90% and traces of other salts (gypsum, bischofite, langbeinite, dolomite, calcite, quartz and sylvine) [41].

Conversion from electrical conductivity (EC), in mS·cm^−1^, to concentration (c), in g·L^−1^, was made using Equation (A1), derived from data tabulated for NaCl aqueous solutions at 20 °C [42,43], as presented in Figure A1.

(A1) $$C=a_{1}\cdot \mathrm{EC}+a_{2}\cdot {\mathrm{EC}}^{2}+a_{3}\cdot {\mathrm{EC}}^{3}+a_{4}\cdot {\mathrm{EC}}^{4}$$

where a_1_ = 0.5135; a_2_ = 7.3802 × 10^−3^; a_3_ = −6.5443 × 10^−5^; a_4_ = 2.1907 × 10^−7^. The maximum error of the correlation is 2.8% (0–300 g·L^−^^1^), and R^2^ = 0.9994.

The correlation was chosen for NaCl aqueous solutions because that for seawater was not available for high salt concentrations. A comparison of this correlation with that for seawater resulted in errors lower than 4%. Since the concentration of NaCl in marine salts is larger than in seawater, it is expected that the error will be lower.
